# Meta-analysis of vitamin D supplementation and hemoglobin concentration: methodological faults obscure the interpretation of the data

**DOI:** 10.1186/s12937-021-00681-w

**Published:** 2021-03-19

**Authors:** Alireza Zimorovat, Mohsen Mohammadi-Sartang, Reza Barati-Boldaji, Hamidreza Raeisi-Dehkordi

**Affiliations:** 1 Nutrition and Food Security Research Center, Shahid Sadoughi University of Medical Sciences, Yazd, Iran; 2 Department of Nutrition, School of Public Health, Shahid Sadoughi University of Medical Sciences, Yazd, Iran; 3 Nutrition Research Center, Shiraz University of Medical Sciences, Shiraz, Iran

**Keywords:** Vitamin D, Hemoglobin, Iron status, Anemia, Letter to the editor

## Abstract

We read the review by Arabi et al. with great interest which tried to examine the effects of vitamin D supplementation on hemoglobin concentration. It seems that the article suffers from fundamental methodological issues and the conclusions are likely to be erroneous. In this regard, we would like to ask the authors to address the mentioned limitations and to update the analysis in order to provide robust and trustful results. We are concerned that such meta-analyses may lead to the biased findings and conclusions.

Dear Editor,

We carefully read the article by Arabi et al. [1] published in Nutrition journal. While applauding the authors’ work, some methodological errors captured our attention, the unclear and controversial inclusion criteria, redundant effect sizes, and inappropriate combination of different effect sizes together  that might lead to biased results.

The authors did not follow the PRISMA guidelines, which focuses on the accuracy and reliability of the reporting of the systematic reviews and meta-analyses. The authors did not sufficiently follow the PICO format (Participants, Intervention, Comparison, and Outcomes). The authors did not clearly address two of the main components of inclusion criteria (participants and comparison). Conducting a systematic review with vague inclusion criteria may lead to problems concerning the validity and applicability of the systematic review [2]. In addition, the registration code or web address of the published protocol has not been provided in the study. Registration of protocol reduces the impact of authors’ biases, reduces the potential for duplication, promotes the transparency of methods and processes, and allows peer reviews of the planned methods [3].

Additionally, we noticed that there are some contradictions in the inclusion criteria. The authors stated that they did not limit their search strategy based on gender, age, etc. (**inclusion criteria, point number 1**), however, they declared further that the studies carried out in subjects with mean age of ≥17.5 years old were included (**inclusion criteria, point number 3**). Also, it is stated that studies which used oral vitamin D supplementation were included (**inclusion criteria, point number 2**), nevertheless, we noticed that they included studies which have used parenteral or enteral vitamin D supplementation. For instance, they included the study done by Sooragonda et al. [4] which administered parenteral vitamin D supplementation for subjects with concurrent iron-deficiency anemia and vitamin D deficiency. Moreover, one study which used enteral vitamin D supplementation for critically ill patients [5] was also included in the meta-analysis which is against their inclusion criteria as well.

Based on the Cochrane handbook, including multiple comparisons from one study with a shared intervention group to the meta-analysis is not correct [6]. This approach double-counts the participants in the shared intervention group, and creates a unit-of-analysis error due to the unaddressed correlation between the estimated intervention effects from several comparisons. The authors included 4 effect sizes from Toxqui et al. study [7] with a shared control group. In fact, they included endpoint assessments in different period (4 weeks, 8 weeks, 12 weeks and 16 weeks from baseline) as different effect sizes, which is not correct. In another similar fault, three different effect sizes (endpoint assessment at week 1, 4 and 6 after the supplementation) were considered as different effect sizes from a same population [8]. Additionally, they included two different effect sizes from one study which assessed the effect of calcium phosphate and vitamin D3 with a shared control group [9]. Two different dosages of vitamin D supplementation (50,000 IU D3, and 100,000 IU D3 daily) were considered as two different effect sizes from a study with a shared placebo group [5], which is erroneous as well. Altogether, the authors included 7 redundant effect sizes.

Lastly, we noticed that the authors combined the effect sizes of co-intervention studies with vitamin D intervention studies, which is not methodologically correct. The authors included a study which assessed the combination effects of food products fortified with vitamin D and calcium in young adults [9]. In addition, one study which assessed the combination effect of vitamin D and vitamin K on healthy trained endurance athletes in an acute phase (endpoints were assessed at baseline and 3-h after the exercise) [10] was also included in the meta-analysis. According to the Cochrane handbook [11], co-intervention studies can be included in the meta-analysis in special cases where the same supplementary intervention is delivered to both intervention and control groups. In other words, the supplementary intervention should not interact (leading to larger (synergistic) or smaller (dysynergistic/antagonistic) effects) with the effects of intervention of interest alone. Thus, the two mentioned studies have to be included in the systematic review but not meta-analysis. How to ensure that the effect on hemoglobin concentration was from vitamin D supplementation, and not from other proposed interventions? Besides, the latter study [10] was performed to assess the acute phase of vitamin D supplementation and should not be combined to the chronic effect sizes according to Cochrane handbook [11].

Therefore, we conclude that the current review has the potential for producing incorrect and misleading results. Hence, the results of the current review should be interpreted with caution because of the mentioned errors. The correction of the stated faults may result in substantial differences in the conclusions that can be drawn from the present meta-analysis.

## Data Availability

Not applicable.

## References

[1] Arabi SM, Ranjbar G, Bahrami LS, Vafa M, Norouzy A (2020). The effect of vitamin D supplementation on hemoglobin concentration: a systematic review and meta-analysis. Nutr J.

[2] Liberati A, Altman DG, Tetzlaff J, Mulrow C, Gøtzsche PC, Ioannidis JP (2009). The PRISMA statement for reporting systematic reviews and meta-analyses of studies that evaluate health care interventions: explanation and elaboration. PLoS Med.

[3] Shamseer L, Moher D, Clarke M, Ghersi D, Liberati A, Petticrew M (2015). Preferred reporting items for systematic review and meta-analysis protocols (PRISMA-P) 2015: elaboration and explanation. Bmj..

[4] Sooragonda B, Bhadada SK, Shah VN, Malhotra P, Ahluwalia J, Sachdeva N (2015). Effect of vitamin D replacement on hemoglobin concentration in subjects with concurrent iron-deficiency anemia and vitamin D deficiency: a randomized, single-blinded, placebo-controlled trial. Acta Haematol.

[5] Smith EM, Jones JL, Han JE, Alvarez JA, Sloan JH, Konrad RJ (2018). High-dose vitamin D3 administration is associated with increases in hemoglobin concentrations in mechanically ventilated critically ill adults: a pilot double-blind, randomized, placebo-controlled trial. J Parenter Enter Nutr.

[6] Cochrane Handbook for Systematic Reviews of Interventions. 16.5.4 How to include multiple groups from one study. Available from: https://handbook-5-1.cochrane.org/chapter_16/16_5_4_how_to_include_multiple_groups_from_one_study.htm. Accessed 27 Dec 2019.

[7] Toxqui L, Pérez-Granados AM, Blanco-Rojo R, Wright I, González-Vizcayno C, Vaquero MP (2013). Effects of an iron or iron and vitamin D–fortified flavored skim milk on iron metabolism: a randomized controlled double-blind trial in iron-deficient women. J Am Coll Nutr.

[8] Panwar B, McCann D, Olbina G, Westerman M, Gutiérrez OM (2018). Effect of calcitriol on serum hepcidin in individuals with chronic kidney disease: a randomized controlled trial. BMC Nephrol.

[9] Trautvetter U, Neef N, Leiterer M, Kiehntopf M, Kratzsch J, Jahreis G (2014). Effect of calcium phosphate and vitamin D 3 supplementation on bone remodelling and metabolism of calcium, phosphorus, magnesium and iron. Nutr J.

[10] Dahlquist DT, Stellingwerff T, Dieter BP, McKenzie DC, Koehle MS (2017). Effects of macro-and micronutrients on exercise-induced hepcidin response in highly trained endurance athletes. Appl Physiol Nutr Metab.

[11] Higgins J, Wells G (2011). Cochrane handbook for systematic reviews of interventions.

